# Motivational climate, need satisfaction, self-determined motivation, and physical activity of students in secondary school physical education in China

**DOI:** 10.1186/s12889-020-09750-x

**Published:** 2020-11-10

**Authors:** Ruzhuan Chen, Lijuan Wang, Bingnan Wang, Yulan Zhou

**Affiliations:** 1 School of Physical Education and Sport Training, Shanghai University of Sport, Shanghai, 200438 China; 2 Physical Education Department, Harbin University of Science and Technology, Rongcheng, 264300 Shandong China

**Keywords:** Self-determined motivation, Physical activity, Need satisfaction, Motivational climate

## Abstract

**Background:**

On the basis of the integration constructs from self-determination theory (SDT) and achievement goal theory (AGT), this study aims to investigate the relationship among classroom motivational climate from four perspectives (i.e. autonomy support, relatedness support, task-involving climate and ego-involving climate), three psychological needs (i.e. autonomy, competence and relatedness), self-determined motivation and physical activity (PA) in secondary physical education (PE).

**Methods:**

Participants consisted of 1186 Chinese students aged 11 to 16 years from three secondary schools in Shanghai. Accelerometers were utilized to measure moderate to vigorous physical activity (MVPA). Questionnaires were used to measure SDT variables (i.e. classroom motivational climate, perceived competence, autonomy, relatedness and self-determined motivation). Structural equation modelling (SEM) was adopted to analyse the hypothesised relationship.

**Results:**

SEM analysis revealed that task-involving climate and autonomy support were positively associated with autonomy, relatedness and competence. Relatedness support was positively related with autonomy and relatedness, whereas ego-involving climate was only associated with competence. The three psychological needs positively affected self-determined motivation, and self-determined motivation positively affected the MVPA time of secondary school students in PE lessons.

**Conclusion:**

These findings support a model of motivation that integrates SDT and AGT, provides new insight into understanding MVPA in Chinese PE, and establishes a solid basis for intervention research.

## Background

Numerous national studies have shown that school-aged children and adolescents in China are physically inactive and do not reach 60 min of daily moderate to vigorous physical activity (MVPA) recommended by the World Health Organization [1–4]. Physical education (PE) is important in promoting adolescents’ physical activity (PA) because it provides opportunities for students to engage in MVPA. Moreover, Students learn sport skills and accumulate sports knowledge in PE classes to help them become physically active in school, beyond school and throughout their lives [5, 6]. The United States’ Centers for Disease Control and Prevention and the United Kingdom’s Association for Physical Education suggested that elementary and secondary school students engage in MVPA for 50% of their time in PE class to obtain health benefits [7]. However, studies found that secondary school students from several countries do not meet this recommended time [8–14]. Hollis et al. [14] found that secondary school students engage in MVPA for an average of 40.5% of their time in PE class. Hence, promoting adolescent MVPA engagement in PE has become an imperative task.

Motivation is an individual drive to act, and several studies have reported that the motivation of students in PE drives them to engage in physical activities and develop their habit of PA participation [15–17]. The relationship between the motivation and PA engagement of students in PE must be investigated to provide evidence to develop strategies in promoting students’ PA level in PE classes [6, 18]. Self-determination theory (SDT) [19] and achievement goal theory (AGT) [20] are two social-cognitive theories that are widely used for studying student motivation in the PE setting.

### Theoretical integration of SDT and AGT

SDT [21] is one of the numerous theoretical frameworks that can provide insights into human motivation and psychological development. According to SDT, a continuum of motivation determines behaviour: intrinsic motivation (an individual engages in activity out of interest in the activity itself), extrinsic motivation (an individual performs an activity to obtain desirable and separate outcomes such as rewards, high grades and praise), and amotivation (an individual perceives no association between behaviour and corresponding outcome) [19, 22]. Extrinsic motivation is composed of four behavioural regulations such as external regulation, introjected regulation, identified regulation and integrated regulation which vary in their level of self-determination. External regulation occurs when an individual’s behaviour is governed by the externally controlled contingencies administered by others. Introjected regulation occurs when an individual’s behaviour is controlled by contingent consequences that they administer by themselves. Identified regulation occurs when individuals participate to gain benefits that they consider important (e.g. fitness gains or weight loss). Integrated regulation is the fullest and most complete form of internalisation of extrinsic motivation, in which individuals consider the benefits they have gained consistent with their core values and beliefs [22]. However, researchers suggested that assessing the integrated regulation of adolescents is not needed because they have yet to develop this type of regulation [23, 24]. According to SDT, self-determined forms of motivation, including intrinsic motivation and identified regulation, are associated with positive outcomes, whereas controlling forms of motivation, such as introjected regulation, external regulation and amotivation, are related to negative outcomes [22]. Another core tenet of SDT is that individuals have innate needs to be competent (i.e. the ability to achieve desired outcomes effectively), autonomous (i.e. having the liberty to make individual choices and a sense of freedom before taking action) and socially related (i.e. connection with and acceptance by significant others). The achievement of these needs can promote self-determined motivation and further influence their cognitive, affective and behavioural consequences [19, 21]. A third fundamental tenet of SDT is that different social contexts either satisfy or thwart the three innate psycholgoical needs for autonomy, competence and relatedness. Under PE context, studies found that perceived need support (autonomy support, competence support, and relatedness support) from PE teachers [25–31] positively affects three types of psychological needs to foster self-determined motivation. Moreover, these three types of needs are positively associated with self-determined motivation and further enhanced their MVPA level [25–27].

Another prevailing theory is AGT, which describes two factors associated with adolescent motivation, namely, individual’s goal orientation (i.e. individuals’ competence towards an activity) and social environment (i.e. motivational climate) [20, 32]. In this study, we only adopted the construct of motivational climate, which refers to a psychological environment that directs students’ competence towards activities in achievement situation in PE [32–34]. According to AGT, motivational climate is divided typically into two climates, namely, task-involving and ego-involving climate. Task-involving climate is characterised by self-references, mistake as part of integral learning, cooperation, effort and task-mastery, whereas ego-involving climate is characterised by normative comparison, less effort and competition with others [34]. Under PE context, a task-involving climate created by teachers is positively related to the satisfaction of the three psychological needs, whereas the ego-involving climate is not related or negatively related to autonomy, competence and relatedness [35, 36].

**Fig. 1:**
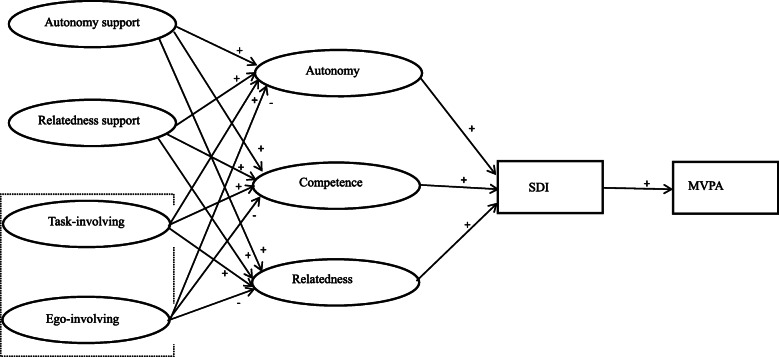
Hypothesized model of motivational process in PE. Note. SDI = self-determined motivation index; MVPA = moderate-to-vigorous physical activity; The components in the dotted box stem from AGT, other components stem from SDT

Both theories emphasise the classroom climate related to support competence. However, SDT does not divide the competence support climate into task-involving and ego-involving climate, which are crucial elements in motivating students in PE [32, 37]. Studies suggested that the two models should be integrated to provide a comprehensive understanding of the motivational mechanisms involved in class [24, 32]. Therefore, the present study integrated the SDT and AGT to investigate the relationship among the four types of PE climate (i.e. students’ perception of autonomy support, task-involving PE climate, ego-involving climate and relatedness support), psychological needs (i.e. autonomy, competence and relatedness), self-determined motivation and MVPA time of students in PE. On the basis of the literature, a hypothesised model was proposed (Fig. 1): (1) self-determined motivation is positively associated with student MVPA in PE. (2) Self-determined motivation can be positively predicted by the satisfaction of the three basic psychological needs. (3) Students’ perceived autonomy support, task-involving PE climate, and relatedness support are positively related to the satisfaction of the three psychological needs, whereas perceived ego-involving climate is negatively related. (4) The three basic psychological needs and self-determined motivation mediate the relationship between the PE climate created by PE teachers and the MVPA of adolescent students in PE.

## Method

### Participants and setting

The current study was approved by ethics committee of Shanghai University of Sport and relevant educational authorities. For convenience, three secondary schools were selected from Shanghai, a city in the eastern part of China. Secondary school has four grades (i.e. Grades 6 to 9) and three or four classes were selected randomly from each grade. A total of 1344 students from 42 classes were invited to participate in this study, and consent forms were sent to them and their parents. Of the 1344 students, 1221 volunteered to participate, with 1199 students providing MVPA and survey data (98.2% response rate).

Participants were taught a 40-min co-educational PE class on alternate days by 18 certified PE teachers with 8 to 15 years of teaching experience in school settings. Data were collected from 42 different PE classes, including three track and field lessons, five game lessons, three aerobics lessons, six basketball lessons, five football lessons, four table tennis lessons, six volleyball lessons, three badminton lessons, three kung fu lessons, and four jump rope lessons. In a typical PE classes, a 5- to 10- min warm-up was provided at the beginning of the class. Then the teachers instructed sports skills and organized students to practice during the middle period of the class (25 to 30 min). Finally, teachers provided cool-down activities and concluded the lesson (5 min).

### Measures

#### MVPA in PE

Actigraph GT3X accelerometers were used to measure MVPA of secondary school students in PE, which had been confirmed to be a valid instrument in measuring the PA of children and adolescents [38, 39]. A one-second epoch was used to avoid underestimating short periods of high-intensity activity; specific cut-off points for Chinese children and youths aged 9 to 17 years were used to determine activity level thresholds, defining MVPA as counts per minute ≥2800 [40].

#### Self-determined motivation

Motivation in PE was assessed using the Perceived Locus of Causality (PLOC) scale (Table 1) [41]. The PLOC included four dimensions (intrinsic motivation, identified motivation, external motivation, and amotivation), each with four items. Evidence on internal consistency and construct validity was found adequate in previous studies [41, 42]. To examine student self-determined motivational levels [36], a self-determination index (SDI) was adopted by using the formula, SDI = (2 × intrinsic motivation) + (identified motivation) – (external motivation) – (2× amotivation) [12, 17].

**Table 1:** MCPES, Basic Psychological Needs and PLOC Scales

Title	Dimension	Items							Liket item						
							1	2	3		4			5	
							Not true	A little true	Sort of true		True			Very true	
MCPES	Autonomy supporting	Item 1. Students have a significant role in decision making in PE lessons							1	2	3	4	5		
		Item 2. Students are given the opportunity to affect the way PE lessons are run							1	2	3	4	5		
		Item 3. Students have significant freedom to make choices during PE lessons							1	2	3	4	5		
		Item 4. Students are given the opportunity to select activities according to their own interests							1	2	3	4	5		
		Item 5. Students can affect the course of PE lessons							1	2	3	4	5		
	Relatedness supporting	Item 6. we have a good sense of unity in PE class							1	2	3	4	5		
		Item 7. Our PE class is united when practicing during PE lessons							1	2	3	4	5		
		Item 8. Students really “work together” as a team							1	2	3	4	5		
		Item 9. During PE lessons the students “pull together”							1	2	3	4	5		
	Task-involving	Item 10. It is important for the students to try their best during PE lessons							1	2	3	4	5		
		Item 11. Learning new things makes me want to learn more							1	2	3	4	5		
		Item 12. What’s most important is that we progress every year in our motor skills							1	2	3	4	5		
		Item 13. It is important for the students to try to improve their own skills							1	2	3	4	5		
		Item 14. It is important to keep trying even though you make mistakes							1	2	3	4	5		
	Ego-involving	Item 15. It is important for students to show that they are better in PE than others							1	2	3	4	5		
		Item 16. During PE lessons students compare their performance mainly to that of others							1	2	3	4	5		
		Item 17. It is important for the students to succeed better than the others							1	2	3	4	5		
		Item 18. During PE lessons the students compete with each other in their performance							1	2	3	4	5		
Basic Psychological Needs	Autonomy	Item 1. I have time to create my own game/dances.							1	2	3	4	5		
		Item 2. I have time to choose my partner.							1	2	3	4	5		
		Item 3. I have time to choose which activities I want to practice.							1	2	3	4	5		
	Competence	Item 4. I think I am good at PE.							1	2	3	4	5		
		Item 5. I feel I am good in sport skills.							1	2	3	4	5		
		Item 6. I feel I am able to do most of games well in PE							1	2	3	4	5		
	Relatedness	Item 7. I feel I am encouraged by other students in PE class							1	2	3	4	5		
		Item 8. I feel comfortable with my partner/team members in PE class							1	2	3	4	5		
		Item 9. I feel I am important to my classmates in PE class.							1	2	3	4	5		
			1	2		3	4	5		6			7		
			Corresponds not all	Corresponds a very little		Corresponds a little	Corresponds moderately	Corresponds enough		Corresponds a lot			Corresponds exactly		
PLOC	Intrinsic motivation	Item 1. Because PE is fun							1	2	3	4	5	6	7
		Item 2. Because I enjoy learning new skills							1	2	3	4	5	6	7
		Item 3. Because PE is exciting							1	2	3	4	5	6	7
		Item 4. Because of the enjoyment that I feel while learning new skills/techniques							1	2	3	4	5	6	7
	Identified motivation	Item 5. Because I want to learn sport skills							1	2	3	4	5	6	7
		Item 6. Because it is important for me to do well in PE							1	2	3	4	5	6	7
		Item 7. Because I want to improve in sport							1	2	3	4	5	6	7
		Item 8. Because I can learn skills which I could use in other areas of my life							1	2	3	4	5	6	7
	External motivation	Item 9. Because I’ll get into trouble if I don’t							1	2	3	4	5	6	7
		Item 10. Because that’s what I am supposed to do							1	2	3	4	5	6	7
		Item 11. So that the teacher won’t yell at me							1	2	3	4	5	6	7
		Item 12. Because that’s the rule							1	2	3	4	5	6	7
	Amotivation	Item 13. But I really don’t know why							1	2	3	4	5	6	7
		Item 14. But I don’t see why we should have PE							1	2	3	4	5	6	7
		Item 15. But I really feel I’m wasting my time in PE							1	2	3	4	5	6	7
		Item 16. But I can’t see what I’m getting out of PE							1	2	3	4	5	6	7

*MCPES* Motivational Climate in Physical Education Scale, *PLOC* Perceived Locus of Causality

#### Basic psychological needs in physical education

The Psychological Needs Satisfaction Scale (Table 1) in PE lesson [43] was adopted to measure the autonomy, competence, and relatedness of students. Each subscale of the three psychological needs had three items, resulting in a total of nine items. The composite reliability coefficients for autonomy, competence, and relatedness were acceptable [43].

#### Motivational climate in physical education

Students’ perception of the motivational climate in PE were assessed using the Motivation Climate in Physical Education Scale (MCPES) [44], which consists of four subscales that measure the climate of autonomy support, relatedness support, task-involving, and ego-involving (Table 1). The scale has 18 items, with five items assessing autonomy support, five items assessing task-involving climate, four items assessing relatedness support and four items assessing ego-involving climate. Internal consistency and construct validity of the sample of secondary school students were adequate [32]. Table 1 shows the three scales.

#### Translation procedures

Before data collection, questionnaires were translated into Chinese and validated. Translation and back-translation of the scales were undertaken by two experts who are fluent in both Chinese and English. The back-translated version was then compared with the original version and noted differences were negotiated by the two translators until the translators agreed. The Chinese version of the questionnaire was sent to five experts to verify the validity, and modifications were made on the basis of suggestions from the experts. For instance, the item ‘what’s most important is that we progress every year in our skill’ was changed to ‘what’s most important is that we progress every year in our motor skills’. The item ‘our PE class has a good sense of unity’ was changed to ‘we have a good sense of unity in PE class’ because the new item was more in line with the language habits of Chinese adolescents.

### Data collection

Data were collected by the first author and three research assistants from December 2018 to January 2019. Prior to starting each PE class, the research purpose was explained, and instructions on how to wear the accelerometers were provided. Before the beginning of a typical PE class, participating students were asked to wear the Actigraph GT3X accelerometers. The accelerometers were fastened to the right hipbone by an elastic belt to secure the device for the entire lesson. A research assistant monitored the students to ensure that they did not remove the accelerometers during the PE classes. All accelerometers were returned by the end of the class. Therefore, the valid duration of wearing an accelerometer was defined as 100% of PE class time. After class, the Actigraph data were downloaded to individual computers via the ActiLife software 6.11.5, and raw accelerometer counts were converted into minutes spent in MVPA per class. After class, printed questionnaires were distributed to the students. The survey was completed within approximately 15 min. The questionnaires were collected immediately upon completion. Research assistants carefully checked each questionnaire if any items were missed. If some items were missed, then the participant student was required to take it back and complete it.

### Data analysis

Data was anlysed using IBM SPSS Amos 21.0 in this study. Confirmatory factor analysis (CFA) was initially employed to estimate the adequacy of the measurement model. Structural equation modelling (SEM) analysis with maximum likelihood estimation was conducted to examine whether the hypothesised theoretical model specified in Fig. 1 fit the data in this study. Model fit to the data was inspected using chi-square statistic (X^2^) value, goodness-of-fit index (GFI), incremental fit index (IFI), comparative fit index (CFI), standardised root square residual (SRMR) and root mean square error of approximation (RMSEA). According to Hu and Bentler [45], CFI and IFI with values close to or greater than 0.90 and RMSEA and SRMR with values of 0.06 and 0.08 or less, respectively, show an acceptable model fit. This study also used bootstrap-generated bias-corrected confidence approach to explore the mediated relationship among variables.

## Results

### Demographic characteristics

Out of the 1199 participants, 11 students provided incomplete data, and two students were outliers because of accelerometer malfunction. All these data were consequently eliminated because they could potentially bias the results. Hence, the final analytic sample consisted of 1186 participating students. The ages of participants ranged from 11 to 16 years (M = 13.09, SD =1.38). The break down by gender was 538 (45.4%) boys and 648 (54.6%) girls. Participants consisted of 318 (26.8%) grade six students, 306 (25.8%) grade seven students, 259 (21.8%) grade eight students, and 303 (25.5%) grade nine students (Table 2).

**Table 2:** Demographic characteristic and Descriptive results

Title	Dimension	N	Percentage	M	SD
Demographic characteristic	Gender				
	Boy	538	45.4%		
	Girl	648	54.6%		
	Grade				
	Grade Six	318	26.8%		
	Grade Seven	306	25.8%		
	Grade Eight	259	21.8%		
	Grade Nine	303	25.5%		
	Age			13.09	1.38
Four types of classroom climates	Autonomy support			3.74	0.98
	Relatedness support			4.43	0.80
	Task-involving			4.45	0.67
	Ego-involving			3.21	1.07
Basic psychological needs	Autonomy			3.75	0.86
	Competence			3.84	0.93
	Relatedness			4.08	0.85
Motivation	Intrinsic motivation			5.68	1.36
	Identified motivation			5.84	1.24
	External motivation			2.84	1.58
	Amotivation			1.44	1.14
	MVPA			15.5(min)	4.61
	MVPA%			38.8	11.5

*N* number, *M* mean, *SD* standard deviation, *MVPA* moderate-to-vigorous physical activity

### Scale reliability and validity

Mardia’s multivariate kurtosis coefficient was used to examine the normality of each variable, and the results indicated that the data distribution was abnormal. CFA was implemented to test the construct validity of scales. CFA was conducted by using the maximum likelihood estimation method with the bootstrapping procedure, because it does not require a normal distribution of data. The measurement model for all the scales shows an acceptable fit to the observed data (X^2^ = 3626.927, df = 805, *P* < .0001; CFI = 0.916; IFI = 0.916; SRMR = 0.053; RMSEA = 0.054). Factor loadings of all observed variables ranged from 0.281 to 0.939, with most of them exceeding 0.4 except for two items (i.e. ‘because that’s what I am supposed to do,’ with factor loading of 0.281 and ‘because I can learn skills which I could use in other areas of my life’ with factor loading of 0.341). When these two items were excluded from the scales, the CFA results showed that the fit index of CFA improved (X^2^ = 3113.877, df = 724, *P* < .0001; CFI = 0.928; IFI = 0.928; SRMR = 0.0509; RMSEA = 0.053). The factor loadings of all observed variables ranged from 0.527 to 0.939.

The test–retest reliability was checked with 34 adolescents between the ages of 12 and 14 for 2 weeks. The text-retest coefficients of most subscales exceed 0.70 and demonstrated acceptable test-retest reliability [46] at 0.82 for autonomy support, 0.73 for social relatedness support, 0.74 for task-involving climate, 0.73 for ego-involving climate, 0.71 for autonomy, 0.75 for competence, 0.78 for relatedness, 0.77 for intrinsic motivation, 0.79 for external motivation, and 0.85 for amotivation. The identified motivation subscale of PLOC exhibits test-retest reliability coefficient of 0.65, which is below 0.70, but the subscale was retained because of its theoretical importance. Cronbach’s alpha coefficient was used to determine internal consistency. Results showed that Cronbach’s alpha coefficients of all scales exceed the acceptable value of 0.70 [47] (see Table 3). These results indicated acceptable internal consistency of all the subscales.

**Table 3:** Internal consistency and correlationship among variables (*N* = 1186)

	1	2	3	4	5	6	7	8	9	10	11
1.Autonomy support	(0.90)										
2.Relatedness support	.48^**^	(0.77)									
3.Task-involving	.42^**^	.61^**^	(0.87)								
4.Ego-involving	.24^**^	.10^**^	.23^**^	(0.86)							
5.Autonomy	.57^**^	.42^**^	.42^**^	.23^**^	(0.71)						
6.Competence	.37^**^	.39^**^	.45^**^	.26^**^	.48^**^	(0.72)					
7.Relatedness	.42^**^	.56^**^	.52^**^	.26^**^	.54^**^	.58^**^	(0.73)				
8.Intrinsic motivation	.36^**^	.41^**^	.50^**^	.27^**^	.42^**^	.53^**^	.49^**^	(0.84)			
9.Identified motivation	.31^**^	.46^**^	.53^**^	.12^**^	.36^**^	.41^**^	.46^**^	.63^**^	(0.76)		
10.External motivation	−.09^**^	−.15^**^	−.11^**^	.08^**^	−.12^**^	−.13^**^	−.09^**^	−.08^**^	−.03	(0.72)	
11.Amotivation	.09^**^	−.20^**^	−.29^**^	.06^*^	−.17^**^	−.17^**^	−.19^**^	−.21^**^	−.27^**^	.43^**^	(0.92)
12.MVPA	−.07^*^	−.02	.08^*^	.00	.01	.15^**^	.05	.16^**^	.12^**^	−.03	−.12^**^

Internal consistency is provided along the diagonal. MVPA = moderate-to-vigorous physical activity

^*^P < .05, ^**^P < .01

### Descriptive analysis and bivariate correlations

Table 2 presents the descriptive statistics and bivariate correlation for the scales. Except for external motivation (M = 2.84, SD = 1.58) and amotivation (M = 1.44, SD = 1.14), the mean scores of other variables were higher than the midpoint. Students spent an average of 15.5 min in MVPA, accounting for 38.8% of the duration of the PE lesson.

Given that the present data indicated abnormal distribution, the bivariate correlation for each variable was calculated by using Spearman’s correlation. Bivariate correlation results showed that the four types of classroom climate variables, namely autonomy support, relatedness support, task-involving climate and ego-involving climate, were positively related to autonomy (*ρ =* 0.57, *P* < 0.01; *ρ =* 0.42, *P* < 0.01; *ρ =* 0.42, *P <* 0.01;*ρ =* 0.23, *P* < 0.01), competence (*ρ =* 0.37, *P* < 0.01; *ρ =* 0.39, *P <* 0.01; *ρ =* 0.45, *P <* 0.01; *ρ =* 0.26, *P <* 0.01) and relatedness (*ρ =* 0.42, *P* < 0.01; *ρ =* 0.56, *P* < 0.01; *ρ =* 0.52, *P <* 0.01; *ρ =* 0.26, *P <* 0.01). Autonomy, competence and relatedness were positively related to intrinsic motivation (*ρ =* 0.42, *P <* 0.01; *ρ =* 0.53, *P <* 0.01; *ρ =* 0.49, *P <* 0.01) and identified motivation (*ρ =* 0.36, *P <* 0.01; *ρ =* 0.41, *P <* 0.01; *ρ =* 0.46, *P <* 0.01), but were negatively related to external motivation (*ρ =* − 0.12, *P <* 0.01; *ρ =* − 0.13, *P <* 0.01; *ρ =* − 0.09, *P <* 0.01) and amotivation (*ρ =* − 0.17, *P <* 0.01; *ρ =* − 0.17, *P <* 0.01; *ρ =* − 0.19, *P <* 0.01). Intrinsic motivation and identified motivation were positively associated with student MVPA (*ρ =* 0.16, *P <* 0.01; *ρ =* 0.12, *P <* 0.01) in PE (Table 3).

### Testing hypothesized structural modelling

**Fig. 2:**
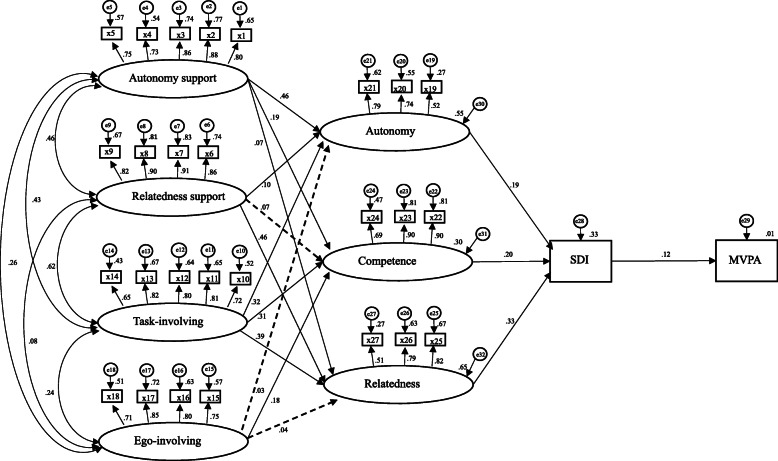
Self-determination process model with standardized coefficients for participants. Note. SDI = self-determined motivation index; MVPA = moderate-to-vigorous physical activity; Broken lines represent nonsignificant standardized parameter estimates; Solid lines represent significant standardized parameter estimates

SEM was used to test the relationship among the latent variables outlined in Fig. 1. The results of path analysis revealed good fit to the data (X^2^ = 2048.997, df = 357, *P <* 0.0001; CFI = 0.918; IFI = 0.918; SRMR = 0.067; RMSEA = 0.058) (Fig. 2). The R^2^ value (Table 4) indicated that 54.8, 64.8 and 30% of the variance in autonomy, relatedness and competence scores were explained by four types of classroom climate variables including autonomy support, relatedness support, and task- and ego-involving climate, respectively. Moreover, 33.2% of the variance in self-determined motivation was predicted by autonomy, competence, and relatedness. Finally, 1.4% of the variance in students’ MVPA in PE was explained by self-determined motivation. Most of the regression weights were significant and positive except for three paths, namely, the path from relatedness support to competence (β = 0.072, *P* > 0.05), the path from ego-involving climate to autonomy (β = 0.028, *P >* 0.05) and the path from ego-involving climate to relatedness (β = 0.042, *P >* 0.05).

**Table 4:** Standardized Parameter Estimates of Indirect Effects and R^2^ value

Parameter	Total effect	Direct effect	Indirect effect
Autonomy support → SDI	0.072^*^	−0.053	0.125^**^
Task-involving climate → SDI	0.542^**^	0.394^**^	0.149^**^
Relatedness support → SDI	0.052	−0.042	0.094^*^
Ego-involving climate → SDI	−0.075^*^	−0.126^**^	0.051^**^
Autonomy support → MVPA	−0.133^**^	−0.166^*^	0.033
Task-involving climate → MVPA	0.177^**^	0.078	0.099^**^
Relatedness support → MVPA	−0.040	−0.006	−0.034
Ego-involving climate → MVPA	−0.006	−0.016	0.009^**^
Relatedness → MVPA	−0.096	−0.118^*^	0.021
Autonomy → MVPA	0.029	−0.001	0.029^**^
Competence → MVPA	0.191^**^	0.181^**^	0.010
	R^2^ value		
Autonomy	0.548		
Competence	0.300		
Relatedness	0.648		
SDI	0.332		
MVPA	0.014		

*SDI* self-determined motivation index, *MVPA* moderate-to-vigorous physical activity

^*^P < .05, ^**^P < .01

To examine the mediating effect, the 11 direct paths presented in Table 4 were added to the hypothesised model. After the 2000 bootstrap samples were extracted from the raw data, the results showed the partial mediation for task-involving climate (β = 0.149, *P* < 0.01; bootstrap 95% CI = 0.093–0.221) to self-determined motivation through autonomy, competence and relatedness. The partial mediation was supported for ego-involving climate (β = 0.051, *P <* 0.01; bootstrap 95% CI = 0.025–0.088) to self-determined motivation only through competence.

## Discussion

The present study aimed to examine the relationship among PE climate (i.e. autonomy support, relatedness support, task-involving climate and ego-involving climate), three basic psychological needs (i.e. autonomy, competence and relatedness), self-determined motivation and MVPA of secondary school students. Findings showed that the autonomy support and task-involving climate students perceived were positively related to all three psychological needs, whereas relatedness support positively predicted autonomy and relatedness, and ego-involving climate was only positively related to competence. Autonomy, competence and relatedness are positively associated with self-determined motivation towards MVPA. Finally, self-determined motivation positively predicted the MVPA of secondary school students.

### Self-determined motivation

Results of the present study indicated that the self-determined motivation of secondary school student was positively related to their MVPA during PE. This finding supported our first hypothesis. This finding was also consistent with the tenet of SDT [22] and previous studies in PE context [15, 17, 48]. However, the findings of the present study showed that self-determined motivation only accounted for 1.4% of the variance in student MVPA. This was lower than the results of previous studies under the PE context, ranging from 2 to 4% [15, 17, 48]. The nature of the Chinese PE curriculum and Chinese educational culture may contribute to the low interpretation rate of variance in student MVPA. Firstly, the Chinese PE curriculum is compulsory, and each student must attend PE classes. Moreover, the class content and PE activities are arranged by secondary schools and teachers, and students cannot choose activities in PE class. Therefore, student motivation is a non-factor in PE classes, possibly resulting in the small contribution of self-determined motivation to student MVPA. Secondly, education in China is based on Confucian principles, which have educated students to be obedient and place emphasis on self-control and personal restraint [49, 50]. This practice may lead to students’ lack of knowledge of their own motivation and interests, thus weakening the effect of their self-determined motivation on their MVPA participation. Although self-determined motivation only explained 1.4% of the variance in student MVPA, it remained important, as students with self-determined motivation were likely to continue to participate in MVPA out of the PE class [26, 27]. On the basis of this finding, PE teachers are advised to adopt instructional strategies (e.g. cooperative learning; Supportive, Active, Autonomous, Fair, Enjoyable [SAAFE] teaching principles [51]) and activities to stimulate students’ interest and improve students’ self-determined motivation, which ultimately enhances MVPA in PE.

### Psychological needs

Results showed that the three psychological needs of autonomy, competence and relatedness positively predicted self-determined motivation. Therefore, when students perceived more competence to exhibit their sport skills, had more opportunities to determine and were more connected with classmates in PE classes they are more likely to be intrinsically motivated to participate in MVPA [24, 27, 29, 31, 35, 36, 52, 53]. Furthermore, the satisfaction of the three psychological needs mediated the relationship between the PE climate created by teachers and self-determined motivation, which was consistent with the tenet of SDT [22]. The importance of the psychological needs of autonomy, competence and relatedness were suggested by the direct and indirect relationships. The fourth hypothesis was partly supported. The findings suggested that PE teachers may provide students with opportunities to cooperate in PE classes (relatedness), give choices to students and involve them in the decision-making process (autonomy) and provide students with more positive experiences for learning and mastering various motor skills (perceived competence) to motivate them to engage in PE activities.

Relatedness was the strongest predictor of self-determined motivation among the three psychological needs in this study. This finding was consistent with the studies of Standage et al. [24] and Cox et al. [53], but it was different from other studies [29, 42, 52] that found perceived competence as the most important variable to self-determined motivation. Deci and Ryan [21, 22] argued that the relative effect of each psychological need satisfaction on self-determined motivation may vary depending on the functional significance of the context. The strongest influence of relatedness may be related to the emphasis of PE on student cooperation in China. The Chinese Curriculum Standard for Physical Education and Health of the primary and middle schools indicated that one of the objectives of the PE curriculum was to develop student social adaption [54]. Pedagogical models that addressed student collaboration (e.g. cooperative learning approach) were recommended to PE teachers to develop students’ collectivism and cooperative consciousness [54]. More opportunities were provided for students to interact with peers in PE classes. These opportunities possibly allowed them to find PE fun and exciting and ultimately encouraged them to participate in PE activities. The low importance of student autonomy and competence in SDT in this study is also understandable because of students’ insufficient autonomy in PE classes and the weakening importance of sports skills and sports performance in the current PE learning assessment system [54].

### Influence of PE climate

The finding of this study showed that autonomy support positively predicted the three psychological needs. Specifically, students felt autonomous, competent, and related when perceiving autonomy support with low control. This finding supported the third hypothesis and was congruent with previous studies in the context of PE [29, 30]. Furthermore, the magnitude of the standardised regression coefficients from autonomy support to the three psychological needs was the highest among the four types of classroom climate, suggesting that autonomy support mostly influenced the satisfaction of the three psychological needs. However, the Chinese traditional teaching style was authoritarian or controlling in PE classes [55]. The mean score of student perceptions of autonomy support climate was lower than that of relatedness support and task-involving climates in our study, thus confirmed this fact.

With regard to relatedness support, research findings indicated that student learning in a PE environment where good relationship among students were established felt more autonomous and related in PE, which was consistent with previous research [53]. Unlike the third hypothesis, the path from relatedness support to student competence was nonsignificant, revealing that the relatedness support PE teachers created could not predict student perception of competence in PE. The possible reason was that the harmonious relationship and emphasis on cooperation among students may weaken competition among students [56], distracting student attention from their competence and performance in PE.

Research findings showed that the student perception of task-involving climate was positively and significantly related to psychological need satisfaction of autonomy, competence and relatedness. Students felt more autonomous, competent, and related when they perceived that PE teachers emphasised their effort and self-reference on success or progress. These findings were aligned with previous study in the context of PE [35] and sports [57]. However, unlike the third hypothesis, the results showed that ego-involving climate was positively associated with competence and not significantly related to autonomy and relatedness. Such climate resulted in competition among students, which may help improve their sports competence [58]. The nonsignificant influence of ego-involving climate on relatedness and autonomy was also accepted because this type of climate does not address student connectedness and autonomy.

Given the importance of motivational climate on autonomy, competence and relatedness, a class climate that focuses on autonomy, competence and relatedness of students is needed to promote student psychological needs. PE teachers should provide autonomy support and are suggested to exert effort in giving students more freedom to make choices, involving them in decision making and respecting their personal volition during PE classes. Relatedness support should be cultivated, and PE teachers should set challenging goals for cooperative groups and emphasise the importance of team progress so as to unite students as a group. Although ego-involving climate contributed to improving students’ perceived competence, its adoption by PE teachers is prudent because it emphasises the normative comparison among students [59]. Finally, PE teachers are suggested to create a task-involving climate that emphasises effort, progress on self-referenced criterion and learning new things during the PE lessons through a variety of teaching strategies and methods (e.g., TARGET approach [33]). Although PE plays a role on increasing MVPA in schools, it is important to note that the main target of PE is to develop children’s movement skills, which might improve movement skill competence and promote engagement in PA during other periods of time and long term [60].

#### Strengths and limitations

The present study has several strengths. Firstly, it was the first to examine the relationship among the PE climate from the four dimensions, the satisfaction of psychological needs, motivation, and accelerometer-determined MVPA in secondary school students. Secondly, objective measures were adopted to evaluate the MVPA of secondary school students to eliminate the bias of subjective scales. Thirdly, the present study provided new insights into how the manners by which students perceive the PE climate created by PE teachers had different influence on autonomy, competence and relatedness. However, this study has limitations. Firstly, this study is cross-sectional; hence, causal inferences cannot be made. Further longitudinal and intervention studies are needed. Secondly, participants were only from three secondary schools in Shanghai, China. Thus, the results of this study may not be used to generalise situations in other populations. Future research should expand the population beyond Shanghai to other regions or countries. The third limitation was the use of the SDT index to assess students’ motivation. Future studies should focus on analysing the relationship among intrinsic motivation, integrated motivation, identified motivation, introjected motivation, external motivation, amotivation and MVPA in PE. Fourthly, this study did not consider the influence of demographic variables such as gender and age when analysing the relationship between SDT variables and MVPA. Future studies should consider the influence of demographic variables.

## Conclusion

The results of this study emphasised the importance of classroom climate created by PE teachers. In addition, the findings supported that a model of motivation, which integrates SDT and AGT, provided new insights into understanding MVPA in PE in the Chinese curriculum. Findings implied that PE climate that focused on support of autonomy (e.g. providing students with the freedom to make choice), relatedness (e.g. emphasizing students’ cooperation) and task-involving climate, which address students’ effort, should be created to promote students’ psychological needs, self-determined motivation and ultimately increased the MVPA of students in PE.

## Data Availability

The datasets used and/or analyzed during the present study are available from the corresponding author (wanglijuan@sus.edu.cn) on reasonable request.

## Abbreviations

MVPA: Moderate-to-vigorous physical activity
PA: Physical activity
PE: Physical education
SDT: Self-determination theory
AGT: Achievement goal theory
CFA: Confirmatory factor analysis
SEM: Structural equation modeling
SDI: Self-determined motivation index

## References

[1] Wang C, Chen P, Zhuang J (2013). A national survey of physical activity and sedentary behavior of Chinese city children and youth using accelerometers. Res Q Exerc Sport.

[2] Chen P (2017). Physical activity, physical fitness, and body mass index in the Chinese child and adolescent populations: an update from the 2016 physical activity and fitness in China—the youth study. J Sport Health Sci.

[3] Liu Y, Tang Y, Cao Z, Zhuang J, Zhu Z, Wu X, Wang L, Cai Y, Zhang J, Chen P (2018). Results from China’s 2018 report card on physical activity for children and youth. J Phys Act Health.

[4] Wang L, Tang Y (2017). Luo J:school and community physical activity characteristics and moderate-to-vigorous physical activity among Chinese school-aged children: a multilevel path model analysis. J Sport Health Sci.

[5] Fairclough S, Stratton G (2005). Improving health-enhancing physical activity in girls' physical education. Health Educ Res.

[6] Hills AP, Dengel DR, Lubans DR (2015). Supporting public health priorities: recommendations for physical education and physical activity promotion in schools. Prog Cardiovasc Dis.

[7] U.S (2010). Department of Health and Human Services, Centers for Disease Control and Prevention, National Center for Chronic Disease Prevention and Health Promotion, Division of Adolescent and School Health. Strategies to improve the quality of physical education.

[8] Ferreira FS, Mota J, Duarte JA (2014). Patterns of physical activity in Portuguese adolescents. Evaluation during physical education classes through accelerometry. Arch Exerc Health Dis.

[9] Dudley DA, Okely AD, Cotton WG (2012). Physical activity levels and movement skill instruction in secondary school physical education. J Sci Med Sport.

[10] Conley MM, Gastin PB, Brown H, Shaw C (2011). Heart rate biofeedback fails to enhance children's ability to identify time spent in moderate to vigorous physical activity. J Sci Med Sport.

[11] Chow BC, Mckenzie TL, Louie L (2009). Physical activity and environmental influences during secondary school physical education. J Teach Phys Educ.

[12] Erwin HE, Stellino MB, Beets MW, Beighle A, Johnson CE (2013). Physical education lesson content and teacher style and elementary students’ motivation and physical activity levels. J Teach Phys Educ.

[13] How YM, Whipp PR, Dimmock JA, Jackson B (2013). The effects of choice on autonomous motivation, perceived autonomy support, and physical activity levels in high school physical education. J Teach Phys Educ.

[14] Hollis JL, Sutherland R, Williams AJ, Campbell E, Nathan N, Wolfenden L, Morgan PJ, Lubans DR, Gillham K, Wiggers J. A systematic review and meta-analysis of moderate-to-vigorous physical activity levels in secondary school physical education lessons. Int J Behav Nutr Phy. 2017;14(1). 10.1186/s12966-017-0504-0. 10.1186/s12966-017-0504-0 PMC5402678 28438171

[15] Ning W, Pope Z, Gao Z (2015). Associations between adolescents situational motivation and objectively-determined physical activity levels in physical education. JTRM Kinesiol.

[16] Chu TLA, Zhang T (2018). Motivational processes in sport education programs among high school students. Eur Phys Educ Rev.

[17] Owen KB, Astell-Burt TPD, Lonsdale CPD (2013). The relationship between self-determined motivation and physical activity in adolescent boys. J Adolesc Health.

[18] Van den Berghe L, Vansteenkiste M, Cardon G, Kirk D, Haerens L (2014). Research on self-determination in physical education: key findings and proposals for future research. Phys Educ Sport Pedagog.

[19] Ryan RM, Deci EL (2000). Self-determination theory and the facilitation of intrinsic motivation, social development, and well-being. AM Psychol.

[20] Nicholls JG. The competitive ethos and democratic education. Teach Coll Rec. 1989.

[21] Deci EL, Ryan RM (1985). The general causality orientations scale: self-determination in personality. J Res Pers.

[22] Deci EL, Ryan RM (2000). The “what” and “why” of goal pursuits: human needs and the self-determination of behavior. Psychol Inq.

[23] Chen W (2014). Psychological needs satisfaction, motivational regulations and physical activity intention among elementary school students. Educ Psychol-UK.

[24] Standage M, Duda JL, Ntoumanis N (2003). A model of contextual motivation in physical education: using constructs from self-determination and achievement goal theories to predict physical activity intentions. J Educ Psychol.

[25] Sebire SJ, Jago R, Fox KR, Edwards MJ, Thompson JL (2013). Testing a self-determination theory model of children’s physical activity motivation: a cross-sectional study. Int J Behav Nutr Phys Act.

[26] Fin G, Baretta E, Moreno-Murcia JA, Nodari RJ (2017). Autonomy support, motivation, satisfaction and physical activity level in physical education class. Universitas Psychologica.

[27] Zhang T, Solmon MA, Kosma M, Carson RL, Gu X (2011). Need support, need satisfaction, intrinsic motivation, and physical activity participation among middle school students. J Teach Phys Educ.

[28] Sanchez-Oliva D, Sanchez-Miguel PA, Leo FM, Kinnafick F, García-Calvo T (2014). Physical education lessons and physical activity intentions within Spanish secondary schools: a self-determination perspective. J Teach Phys Educ.

[29] Standage M, Duda JL, Ntoumanis N (2006). Students' motivational processes and their relationship to teacher ratings in school physical education: a self-determination theory approach. Res Q Exerc Sport.

[30] Standage M, Gillison FB, Ntoumanis N, Treasure DC (2012). Predicting students' physical activity and health-related well-being: a prospective cross-domain investigation of motivation across school physical education and exercise settings. J Sport Exercise Psy.

[31] Standage M, Duda JL, Ntoumanis N (2005). A test of self-determination theory in school physical education. Br J Educ Psychol.

[32] Jaakkola T, Wang CK, Soini M, Liukkonen J (2015). Students’ perceptions of motivational climate and enjoyment in Finnish physical education: a latent profile analysis. J Sports Sci Med.

[33] Ames C, Archer J (1988). Achievement goals in the classroom: Students' learning strategies and motivation processes. J Educ Psychol.

[34] Ames C (1992). Classrooms: goals, structures, and student motivation. J Educ Psychol.

[35] Gråstén A, Watt A (2017). A motivational model of physical education and links to enjoyment, knowledge, performance, Total physical activity and body mass index. J Sports Sci Med.

[36] Ntoumanis N (2005). A prospective study of participation in optional school physical education using a self-determination theory framework. J Educ Psychol.

[37] Abós Catalán Á, Sevil Serrano J, Martín-Albo Lucas J, Julián Clemente JA, García-González L (2018). An integrative framework to validate the need-supportive teaching style scale (NSTSS) in secondary teachers through exploratory structural equation modeling. Contemp Educ Psychol.

[38] Plasqui G, Bonomi AG, Westerterp KR (2013). Daily physical activity assessment with accelerometers: new insights and validation studies. Obes Rev.

[39] Robusto KM, Trost SG (2012). Comparison of three generations of ActiGraph™ activity monitors in children and adolescents. J Sports Sci.

[40] Zhu Z, Chen P, Zhuang J (2013). Intensity classification accuracy of accelerometer-measured physical activities in Chinese children and youth. Res Q Exerc Sport.

[41] Goudas M, Biddle S, Fox K: Perceived locus of causality, goal orientations, and perceived competence in school physical education classes. Br J Educ Psychol 1994; 64 (Pt3) (3):453–463. Doi: 10.1111/j.2044-8279.1994.tb01116.x. 10.1111/j.2044-8279.1994.tb01116.x 7811633

[42] Ntoumanis N (2001). A self-determination approach to the understanding of motivation in physical education. Br J Educ Psychol.

[43] Chen W, Hypnar AJ (2015). Elementary school students’ self-determination in physical education and attitudes toward physical activity. J Teach Phys Educ.

[44] Soini M, Liukkonen J, Watt A, Yli-Piipari S, Jaakkola T (2014). Factorial validity and internal consistency of the motivational climate in physical education scale. J Sports Sci Med.

[45] Hu L, Bentler PM (1999). Cutoff criteria for fit indexes in covariance structure analysis: conventional criteria versus new alternatives. Struct Equ Model Multidiscip J.

[46] Nunnally JC (1994). Bernstein, I: psychometric theory.

[47] Nunnally J (1978). C: psychometric theory.

[48] Aelterman N, Vansteenkiste M, Van Keer H, Van den Berghe L, De Meyer J, Haerens L (2012). Students' objectively measured physical activity levels and engagement as a function of between-class and between-student differences in motivation toward physical education. J Sport Exercise Psy.

[49] Wang L, Zhang Y (2016). An extended version of the theory of planned behaviour: the role of self-efficacy and past behaviour in predicting the physical activity of Chinese adolescents. J Sports Sci.

[50] Au CKF, Johns D, Lindner K (2006). The perceived influence of socialising agents on Hong Kong youth’s entry into sports participation. Physical activity and health of Hong Kong youth.

[51] Lubans DR, Lonsdale C, Cohen K, Eather N, Beauchamp MR, Morgan PJ, Sylvester BD, Smith JJ (2017). Framework for the design and delivery of organized physical activity sessions for children and adolescents: rationale and description of the‘SAAFE’teaching principles. Int J Behav Nutr Phys Act.

[52] Rutten C, Boen F, Vissers N, Seghers J (2015). Changes in Children’s autonomous motivation toward physical education during transition from elementary to secondary school: a self-determination perspective. J Teach Phys Educ.

[53] Cox A, Williams L (2008). The roles of perceived teacher support, motivational climate, and psychological need satisfaction in students' physical education motivation. J Sport Exerc Psychol.

[54] Ministry of Education of the People’s Republic of China (2011). The Chinese Curriculum Standard for Physical Education and Health of the primary and middle schools.

[55] Cheng VMY (2010). Tensions and dilemmas of teachers in creativity reform in a Chinese context. Think Skills Creat.

[56] Cohen J (1982). Cooperative and competitive styles-the construct and its relevance. Hum Relat.

[57] Monteiro D, Teixeira DS, Travassos B, Duarte-Mendes P, Moutão J, Machado S, Cid L. Perceived effort in football athletes: the role of achievement goal theory and self-determination theory. Front Psychol. 2018;9. 10.3389/fpsyg.2018.01575. 10.3389/fpsyg.2018.01575 PMC6121108 30210403

[58] Liukkonen J, Barkoukis V, Watt A, Jaakkola T (2010). Motivational climate and students’ emotional experiences and effort in physical education. J Educ Res.

[59] Jaakkola T, Yli-Piipari S, Barkoukis V, Liukkonen J (2017). Relationships among perceived motivational climate, motivational regulations, enjoyment, and PA participation among Finnish physical education students. Int J Sport Exerc Psychol.

[60] Stodden DF, Goodway JD, Langendorfer SJ, Roberton MA, Rudisill ME, Garcia C, Garcia LE (2008). A developmental perspective on the role of motor skill competence in physical activity: an emergent relationship. QUEST.

